# Is Nutrient Quality of the Locally-Existing, EAT-*Lancet*-like Plant-Based Diet Better or Worse than the Average Diet in Taiwan? An Example of Local Translation

**DOI:** 10.3390/nu16162775

**Published:** 2024-08-20

**Authors:** Wen-Harn Pan, Szu-Yun Wu, Po-Chen Chang

**Affiliations:** 1 College of Public Health, Taipei Medical University, 10F Biomedical Technology Building, No. 301, Yuantong Road, Zhonghe District, New Taipei City 235, Taiwan; 2 Institute of Biomedical Sciences, Academia Sinica, No. 128, Sec. 2, Academia Road, Nankang District, Taipei 115, Taiwan; s.wu@ibms.sinica.edu.tw (S.-Y.W.); jam88430es18@gmail.com (P.-C.C.); 3 Research Center for Environmental Changes, Academia Sinica, No. 128, Sec. 2, Academia Road, Nankang District, Taipei 115, Taiwan; 4 Institute of Population Health Sciences, National Health Research Institutes, No. 35, Keyan Road, Zhunan Town, Miaoli County 350, Taiwan; 5 Institute of Biochemistry and Biotechnology, National Taiwan University, 4F, No. 81, Chang-Xing St., Taipei 106, Taiwan

**Keywords:** EAT-*Lancet*, plant-based diet, red meat, planetary health diet, Nutrition and Health Survey in Taiwan, daily food guide, zinc, vitamin B12

## Abstract

The EAT-*Lancet* commission advocated a planetary health diet in 2019. Some have raised concerns about its nutrient adequacy. This study used data from recent Nutrition and Health Surveys in Taiwan—from 2017 to 2020 (*n* = 6538)—to assess food intake and nutrient adequacy among three red meat consumption levels (low/medium/high). The low red meat group, whose diet was similar to the EAT-*Lancet* reference, showed significantly higher/better levels of vitamins C and E, calcium, magnesium, sodium, dietary fiber, and the polyunsaturated to saturated fatty acids ratio. However, protein, B vitamins, phosphorus for females, and zinc were slightly compromised, but they were still near or above 100% of the Daily Reference Intakes (DRIs), except for zinc (74~75%). The intake levels of vitamin D, calcium, and dietary fiber in all three groups at times did not reach 70% of the DRIs, but this was more pronounced in the high red meat group compared to the low red meat group. Replacing ultra-processed foods (UPFs) with whole/healthy foods improved levels of zinc, calcium, and dietary fiber, but not vitamin D. Finally, a proposed local planetary health dietary construct was provided, suggesting maintaining the original distribution of the food groups recommended by the Taiwan Food Guide while specifying amounts of protein sources in line with the EAT-*Lancet* principles. The proposed diet, according to our estimation and comparison with Taiwanese DRIs, was nearly perfect in its nutrient composition.

## 1. Introduction

The Earth is confronted with a formidable environmental challenge as the greenhouse effect induces a gradual rise in temperature, altering global weather patterns, hampering crop growth, and jeopardizing food security and human survival [1]. By opting for foods that exert minimal impact on the environment, we can contribute to mitigating the detrimental effects of this phenomenon on ecological systems and thereby safeguard food security and the Earth’s sustainability.

In light of the high carbon footprints and health hazards of beef, certain animal products, and processed foods, the EAT-*Lancet* commission in 2019 advocated a plant-based diet (PBD) that curtails the consumption of red meat and ultra-processed foods (UPFs), aspiring for nations across the globe to embrace such a dietary regimen in order to enhance human health, diminish adverse repercussions on the environment, and safeguard healthy longevity and food security [1]. Since the EAT-*Lancet* Commission proposed the planetary health diet (PHD), several studies have investigated the associations among a variety of PHD indices and multiple outcomes. Significant inverse associations have been found in prospective studies for type 2 diabetes, coronary heart disease, CVD mortality, cancer mortality, and total mortality [2]. A meta-analysis of prospective cohort studies [3] has found that the overall PBD index or healthy PBD index scores were associated with a reduced CVD risk. Inverse associations were also observed for measures of adiposity and cardiometabolic disease risk in some studies [4]. In Taiwan, studies investigating the effects of a PBD on the local population have been scarce. A Chinese prospective study using data from a large-scale health check-up observed a substantial lowered rate of aging in those adopting a healthy PBD [5].

Nonetheless, such a diet has elicited concerns about its potential impact on nutritional adequacy, particularly regarding essential micronutrients that are typically found in higher amounts and in more bioavailable forms in animal-derived foods [6]. With this in mind, we employed dietary data from recent Nutrition and Health Surveys in Taiwan (NAHSIT) to conduct empirical scientific validation, examining whether nutrient intake is adequate for the local populace who have already adopted a diet similar to the EAT-*Lancet* recommendations. In this study, we analyzed the nutrient content of the diet low in red meat. Then, the process was repeated again after most of the UPFs in the diet were replaced with whole foods. Considering that local food cultures and produces vary from region to region, the EAT-*Lancet* recommendations provide weight boundaries for food groups, namely, whole grains, tubers or starchy vegetables, vegetables, fruits, dairy foods, non-dairy protein-rich foods, added fats, and added sugars. We attempted to compare the nutrient quality of the local low red meat consumption dietary pattern with that of the high red meat, with or with or without UPFs, to check the impact of red meat/UPFs on nutrient adequacy, and to show the soundness of a Taiwanese diet which follows local food guides and the EAT-*Lancet* principles.

## 2. Materials and Methods

**Figure 1:**
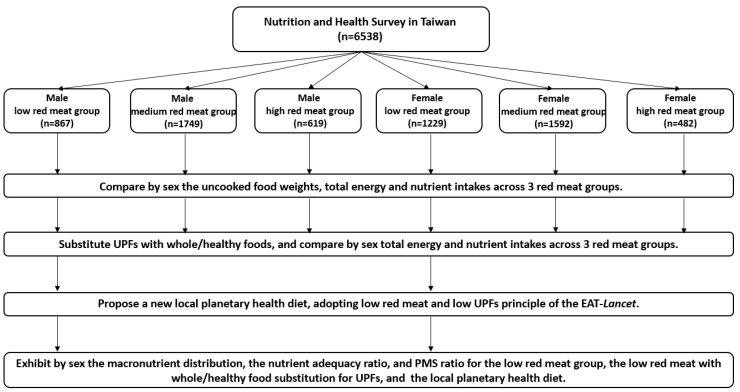
Flowchart of study procedures. PMS ratio, polyunsaturated and monounsaturated to saturated fatty acids ratio; UPFs, ultra-processed foods.

Figure 1 provides a flowchart illustrating all the steps involved in conducting this study.

### 2.1. Data Source

We took advantage of the data from the NAHSIT from 2017 to 2020, a part of the Taiwanese nationwide population-based survey executed on a regular basis. In brief, the target population was non-institutionalized Taiwanese nationals. A stratified, three-stage, clustered sampling scheme was used to ascertain a representative sample of the target population. Details of the survey design and data collection have been published elsewhere [7]. The survey protocol was approved by the Institutional Review Board on Biomedical Science Research, Academia Sinica (project AS-IRB-BM-24004). All participants provided written informed consent prior to the interview. A total of 6538 adult men and women have been included in the statistical analysis. 

### 2.2. Dietary Data from 24-h Recall

Participants’ dietary intake was assessed via 24-h dietary recall in the household interview. The interviewer inquired about the dietary items consumed, the quantity of foods, and cooking recipes with food models designed specifically for the Chinese diet. The 24-h recall information was obtained not only from interviewees, but also from whoever was in charge of cooking. Methods of 24-h recall and nutrient intake computation have been documented previously [8]. Data for total energy, 19 nutrients, and a few dietary components—including 3 macronutrients (carbohydrate, protein, and fat), 9 vitamins (B1, B2, niacin, B6, B12, A, C, D, E), 7 minerals (calcium, phosphorus, iron, zinc, magnesium, potassium, and sodium), 3 groups of fatty acids (saturated, monounsaturated, and polyunsaturated fatty acids), dietary fiber, and cholesterol—were used for statistical analysis in this study.

### 2.3. Defining Local Populace Who Consumed a Low Red Meat Diet

#### 2.3.1. Classification of the Low, Medium, and High Red Meat Groups

The EAT-*Lancet* commission recommended consuming roughly 14 g (or 30 Kcal) of red meat a day for people at the energy level of 2500 Kcal a day [1]. To classify NAHSIT participants into three groups based on red meat consumption levels (low, medium, and high), we first adjusted each individual’s energy intake proportionally to the 2500 Kcal corresponding to the EAT-*Lancet* recommendation. Then, we defined a group of individuals with low red meat consumption: those with an average intake of 30 Kcal from red meat were selected, using thresholds of 92 Kcal for men and 106.5 Kcal for women. Since the Daily Food Guide in Taiwan recommends consuming 7 servings of non-dairy protein-rich foods, including legumes and associated products, aquatic foods, eggs, poultry, and meat, with each contributing roughly 75 Kcal, energy intakes of red meat higher than 525 Kcal (75 Kcal × 7 = 525 Kcal) for both men and women were defined as the ‘high red meat group’. Those with energy intakes of red meat in between the ‘low group’ and the ‘high group’ were defined as the ‘medium group’. Nutrient contents of the diets were compared among the three red meat consumption groups (see Section 2.5).

#### 2.3.2. Substituting UPFs with Whole/Healthy Foods in the Low Red Meat Group to Mimic EAT-Lancet Diet

The EAT-*Lancet* diet restricts the intake of UPFs. In order to ascertain the impacts of UPFs on nutrient adequacy in Taiwanese diets, one-third of both the refined grains and associated products and the UPFs consumed were substituted with the same servings of their whole (or healthier) food counterparts (see Table S1 for food substitution lists), excluding seasonings, condiments, dressings, and sauces, and then nutrient contents were assessed again and compared between the original low red meat diets and the ones with replacement. 

### 2.4. Comparing EAT-Lancet-like Diet with Recommendations Made by Taiwanese Food Guide

To understand the gap between the Taiwanese Food Guide and the EAT-*Lancet* principles, we arranged the food consumption data in the format of the Taiwanese Daily Food Guide, which recommends to the public the ideal serving numbers of the six food groups (grains and roots, dairy, non-dairy protein-rich foods, vegetables, fruits, added fats and oils). For commensurability, we converted the average food weight consumed to an average number of servings for each food category (see Table S2 for the conversion methods), which was then compared with the Taiwanese Daily Food Guide. Since the food guide does not specify how much of each kind of protein foods should be consumed—rather, it states the priority in the following order: legumes, aquatic foods, eggs, poultry and meat—we specified the serving numbers for dairy, legumes, aquatic foods, egg, poultry, red meat, and added sugar as well as for that of plant oil and animal fats, considering the Taiwanese Food Guide and the EAT-*Lancet* recommendations as well as the self-sufficiency rates of local foods; at the same time, we kept the local recommendations for the other food groups (grains and roots, vegetables, fruits, and seeds and nuts). Nutrient qualities were again evaluated for this locally-modified EAT-*Lancet* diet given the described food distribution in each food group.

### 2.5. Statistical Analysis

Taiwanese food composition tables were used to estimate the nutrient content of the three diets described above: the low red meat diet, the low red meat diet with UPFs substituted with whole/healthy foods, and the Taiwanese EAT-*Lancet* diet. The proportion of calories derived from the three macronutrients, as well as the nutrient adequacy ratio—defined as the mean percentage of Daily Reference Intakes (DRIs) for each micronutrient—were estimated. 

Data distribution and normality were initially checked via Kolmogorov–Smirnov tests and transformed whenever necessary prior to statistical analysis. After transformation, they were rechecked via the same test and assisted by visualization. Log transformation was used for skewed body mass index (BMI) data. Cube root transformation was used for skewed dietary variables. All analyses were weighted and adjusted to obtain population-representative estimates via SUDAAN software (version 11.0.1; RTI International, Research Triangle Park, NC, USA) to account for the complex survey design. Within each sex group, a trend test was performed—with a generalized linear model for continuous variables or a chi-square test for categorical variables—to examine whether the following dietary characteristics had an ordered relationship to levels of red meat: (1) non-cooked food weights of the six food groups, (2) total energy intake, energy contribution from macronutrients, saturated fatty acids (SFAs), and added sugar, and (3) the nutrient adequacy ratio and the polyunsaturated and monounsaturated to saturated fatty acid (PMS) ratio. Age was adjusted in the models wherever appropriate. Residual analyses were conducted to check the normality assumptions of the model for the continuous variables.

## 3. Results

### 3.1. Characteristics of the Three Red Meat Groups

Taiwanese men and women aged 19 or above were classified into three red meat groups (low, medium, and high). The weighted mean energy intakes from red meat were around 25.0 Kcal, 276 Kcal, and 743 Kcal for the low, medium, and high groups, respectively, in men, and around 20.7 Kcal, 200 Kcal, and 579 Kcal in women. Table 1 shows the characteristics of each group.

Overall, the mean BMI of the male participants is in the overweight range and that of the female participants is within the normal weight range across all three red meat groups, with no significant differences observed. The high red meat group tends to be younger and has higher proportion of college graduates. Female high red meat consumers are less likely among the divorced, separated, and widower categories, while their male counterparts tended to have more current smokers. 

### 3.2. Macronutrient Composition and Mean Micronutrient Intake Levels by Three Red Meat Groups

Table 2 shows the mean uncooked food weight of each food group by red meat consumption status. The results indicated that the major red meat consumed in Taiwan was pork. Intake weights of beef and lamb together were around one-third (women) to one-fourth (men) of the pork. With decreased red meat consumption, the intake amount of poultry became significantly higher in both men and women (*P*s for trend = 0.014 and < 0.0001, respectively). Similar increasing trends were also found in a sex-specific manner in “rice, wheat, and other grains” (*P* for trend < 0.0001 for female), soy (*P* for trend = 0.005 for female), nuts (*P* for trend = 0.014 for female), other vegetables (*P* for trend = 0.016 for male), fruits (*P* for trend = 0.008 for female), and animal fats (*P*s for trend = 0.049 for male). Vegetable oils, however, decreased with the decreased red meat consumption in males (*P* for trend = 0.001 for males). Food weights did not differ by meat group for ‘potatoes, cassava, corn, and other roots’, ‘dry beans, lentils, and peas’, egg; ‘fish and seafood’, peanuts, dairy foods, vegetables, added sugar, or palm oils. 

Table 3 shows the total energy intakes and the percentages of caloric contributions from carbohydrates, fats, and proteins for the three red meat groups. Red meat itself is the major component of the caloric contribution in the high red meat consumption group; the average caloric intakes were steadily higher, the greater the amount of red meat consumed, as were proteins, fats, and saturated fats, but the trend in carbohydrates was reversed (All *P*s for trend < 0.0001 for both males and females). The group that consumed the least red meat had a macronutrient distribution (54~56% energy from carbohydrates, 28~30% from fats and 16% from proteins) closer to what the Taiwanese Daily Food Guide recommended compared to the other two groups. SFAs were within the recommended range at 8~9% of total energy in low red meat groups, but were over 10% for both the medium and high red meat groups. Added sugar was around 6~8% of total caloric intake for all three groups. 

In order to ascertain the impact of UPFs on nutrient adequacy, we further replaced them in the way recommended by the Taiwanese Food Guide (see Section 2.3.2), with their whole/healthy foods counterparts, in part or completely. The percentage of energy from proteins increased to 17~18% and that from carbohydrates reduced slightly to 53~54% after the replacement process. The percentages from fats increased about 0.1~0.2%, but the SFAs percentages decreased further around 0.6 to 0.7%. Added sugar was reduced around 3~4%, reaching 3% of the total energy intake.

Table 4 compares the nutrient adequacy (as % of DRIs) of proteins, vitamins, minerals, dietary fibers, and the PMS ratio among the three red meat groups. With respect to vitamin B2, vitamin A, vitamin D, phosphorus for males, vitamin B6 for females, iron, potassium, and monounsaturated to saturated fatty acids (M/S) ratios, similar levels were consumed by all three groups. Higher levels (% of DRIs) of vitamins C and E, calcium, magnesium, sodium and dietary fiber as well as polyunsaturated to saturated fatty acids (P/S) were observed for the low red meat group. On the other hand, the compromised nutrients were protein, vitamin B1, vitamin B2 for females, niacin, vitamin B6 for males, vitamin B12, phosphorus for females, and zinc. Nonetheless, except for zinc, the intake levels of these compromised nutrients were close to or above 100% of DRIs. Zinc reached 74~75% of DRIs. It is the intake levels of vitamin D, calcium, and dietary fiber that for all three groups sometimes did not reach 70% of DRIs or the recommended levels, but this more so in the high red meat group compared to the low red meat group.

After replacing UPFs in the diet with their whole food counterparts, the intake levels of calcium, zinc, and dietary fiber were all slightly improved. No effect was seen for vitamin D. 

### 3.3. Comparing Taiwanese Diet Low in Red Meat and UPFs with Taiwanese Daily Food Guide and Specifying Future Directions for New Taiwanese Food Guide Adopting EAT-Lancet Principles

According to the eighth edition of Taiwanese DRIs, the average energy needs are 2100/1650 Kcal per day for a 64-kg man aged 31–50 years and a 54-kg woman aged 31–50 years, respectively, with a moderate low level of physical activity. The suggested serving numbers for the six food groups in the Taiwanese Daily Food Guide for the 2100/1650 Kcal levels are listed in the second and fifth columns of Table 5 for comparison. The mean energy intake level for those who consumed a mean of 14~16 g (roughly 30 Kcal) of red meat a day and low UPFs was 1814 Kcal and 1533 Kcal for men and women, respectively. These men and women consumed an average of 2.6 servings of vegetables and 1.7~1.8 servings of fruits a day (Table 5). These intake levels are less than the vegetable recommendation (4 servings for men and 3 servings for women) and the fruit recommendation (3.5 servings for men and 2 servings for women) for the 2100 Kcal level and the 1600~1700 Kcal levels (see the second column and the fifth column in Table 5). We would recommend raising these levels to comply with the Taiwanese Daily Food Guide under discussion. 

Taiwanese people consumed on average around half of a glass of milk a day, which is less than the EAT-*Lancet* recommendation of 250 mL (1 serving), and much less than the Taiwanese guideline of 1.5 servings a day. Hence, we would suggest not consuming over 1 serving a day. As for the grains/starchy foods, non-dairy protein-rich foods, and added fats/oils groups, the mean total serving numbers consumed by these people were close to the Taiwanese Daily Food Guide recommendations. However, the grains and starchy foods commonly consumed are usually refined, and should be substituted to reach the recommendation of at least one-third from whole foods. 

Around 6.1 and 5.0 servings of non-dairy protein-rich foods, including nuts/peanuts, were consumed by men and women, respectively. Within the non-dairy protein food group, major contributors were poultry (2.1 servings), soy + nuts (1.8 servings), aquatic animal foods (1.6 servings), eggs (0.7 servings), and pork (0.4 servings) in men, while in women the order was slightly different: soy + nuts (2.0 servings), poultry (1.4 servings), aquatic animal foods (1.1 servings), eggs (0.7 servings), and pork (0.3 servings). This distribution departs just slightly from the Taiwanese Daily Food Guide recommendation, which stresses first plant proteins, then aquatic foods, eggs, poultry, pork, and beef, in that order. Poultry consumption here seems slightly higher than the EAT-*Lancet* recommendation. Accordingly, in Table 5, we reallocated the food amounts to be in line with both the EAT-*Lancet* and Taiwanese local guidelines.

The majority of fats and oils consumed was from non-tropical plant sources, and slightly less than half was from animal fats (primarily pork fat) or saturated fat-rich plant oil (mainly palm oil). For the future Taiwanese Food Guide, we would lower saturated fats to the lowest possible level.

If adjustments were made to the dietary structure of the low red meat group toward the recommendations of the Taiwanese food guide alongside the above modification (see Table 5), we found nutrient density was further improved (Table 6). The levels of vitamin D (females) and calcium can be raised to around 70% the DRIs.

## 4. Discussion 

### 4.1. Dietary Characteristics of the Taiwanese Low Red Meat Consumption Pattern in Contrast to the High Consumption One

From this investigation, we discovered that, in general, the low red meat dietary pattern had similar meal types and layout of local dishes, but with much less red meat and added cooking oil selected or used in the protein-rich dishes. Although those with the low red meat pattern tended to have slightly more poultry, staples, nuts, soy products, and fruits, they had a much lower caloric intake level than their high red meat counterparts, mainly due to much less consumption of red meat and cooking oil.

### 4.2. Nutrient Profile of the Taiwanese Low Red Meat and UPFs Consumption Dietary Pattern

Compared to the high red meat pattern, the low red meat pattern with low UPFs demonstrates a dietary macronutrient distribution toward moderate levels of fats, proteins, and carbohydrates. Specifically, it includes approximately 53~54% of calories from carbohydrates, 28~30% from fats, and 17~18% from proteins, falling within the acceptable macronutrient distribution range (50~65% calories from carbohydrates, 20~30% from fats, and 10~35% from proteins) recommended by several renowned health organizations, including the National Academy of Medicine (formerly known as the Institute of Medicine) [9]. The pattern also exhibits a desirable fatty acid profile, with SFAs comprising around 8% of total energy intake, and both the P/S and M/S ratios being greater than one. In addition, the low red meat-low UPFs consumption pattern had a better nutrient profile than the high red meat one for vitamins C, D for female, and E, calcium, magnesium, sodium, dietary fiber, and for the P/S ratio and the M/S ratio for females. On the other hand, although this dietary pattern contains relatively lower levels of proteins, B vitamins (vitamin B1, B2 for females, niacin, vitamin B6 for males, and vitamin B12), phosphorus, iron for females, zinc, potassium, and sodium than the high red meat pattern, it can still provide over or near 100% of DRIs for most of these nutrients, with the exception of zinc. The low red meat pattern provides 74~75% of the zinc DRIs, which went up to 84% of DRIs after most UPFs were replaced with whole/healthy foods. Zinc reached up to 90% of DRIs when the dietary composition was further improved according to both the Taiwanese Daily Food Guide and the EAT-*Lancet* principles.

Some nutritionists expressed concerns over potential micronutrient deficiencies of calcium, iron, vitamin B12, and zinc in practicing the PHD [6,10]. A previous NAHSIT report revealed that calcium, vitamin D, and dietary fiber are commonly deficient nutrients in Taiwan [11]. Dietary fiber intake will certainly be improved by adopting the PHD. Poor calcium and vitamin D status is in part due to a very low milk consumption (less than half a glass a day), and to fresh pasteurized milk mostly not being fortified with vitamin D in Taiwan. Our viewpoint on calcium and vitamin D will be discussed later (see Section 4.4). 

With regard to vitamin B12, due to the popularity of fermented foods or condiments such as fermented soy sauce, bean paste, smelly tofu, miso, kimchi, etc., vitamin B12 status in the blood is satisfactory, as depicted in a past NAHSIT [12]. Zinc biochemistry was not been monitored in our past surveys. However, zinc intake levels estimated from 24-h recall are in general in the range of 75~100% of DRIs for various age-sex groups [11]. In the study of Beal, Ortenzi, and Fanzo (2023) [6], they assessed micronutrient adequacies of the EAT-*Lancet* reference diet for folate, vitamins A and B12, calcium, iron, and zinc with the application of both globally representative food composition data and the harmonized nutrient reference values proposed in 2020 [13]. The estimated zinc intake was 78% of the recommended nutrient intakes [4], similar to our findings in the low red meat group (74–75% of DRIs). However, we showed that this may be raised to 84% of DRIs, after UPFs were replaced with whole or healthy foods, and that it may be further raised to 90% if the Taiwanese Food Guides are followed. This finding suggests dietary quality is the key for zinc nutrition.

Previous study using data from the United States NHANES of 2003–2018 observed an inverse association between PBD index score and iron inadequacy [14]. The present study found that iron adequacy levels were not significantly different among three red meat consumption levels before UPFs substitution, but showed a positive relationship between iron adequacy and red meat intake in females after UPFs substitution. Nevertheless, the nutrient adequacy ratio was above 90% of DRIs both before and after UPFs substitution in both sexes in our study, similar to the global figures [6]. Furthermore, a previous Taiwanese dietary pattern study has shown that dietary patterns high in UPFs, such as rice or flour products, fried foods, sugary beverages, and processed foods, contributed to an increased risk of anemia in women [15]. These observational studies might mitigate some concerns over poorer iron statuses in the practicing of the PHD. 

### 4.3. Potential Dietary Practices to Improve Vitamin B12 and Zinc Status

To ensure the nutritional adequacy of the PHD, local plant-based foods or dishes rich in vitamin B12 and zinc should be promoted. For example, green soybeans within their shells (also called edamame) are rich in B-vitamins and minerals and are often seasoned with pepper, garlic, and salt and served as a formal dish or snack. A locally popular “milk fish”, containing a rather high amount of vitamin B12, is served in fried, braised, or boiled form with either noodles, rice, or in soup. Vitamin B12- and zinc-rich oysters, abundantly grown in coastal aquafarms surrounding the Taiwan island, can be served raw, in casseroles, or pan-fried with eggs and green vegetables and drizzled with tomato sauce. These foods and others may be selectively promoted in conjunction with the advocating the planetary health PBD to mitigate potential nutrient deficiency problems.

**Figure 2:**
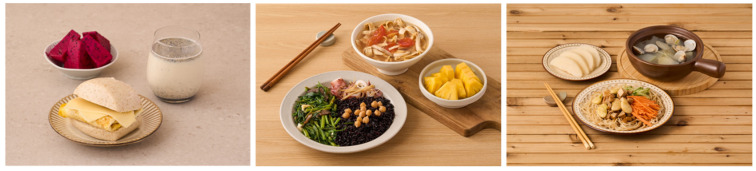
**Suggested plant-based diet plan A at the 2100 Kcal level.** (**Left**) Breakfast: Steamed whole wheat buns with a slice of cheese and a pan-fried egg; Dragon Fruit (red color); Sesame and honey-flavored morning drink with half low-fat milk and half soy bean milk. (**Center**) Lunch: Steamed black rice with chickpeas; Steamed squid; Stir-fried water spinach and black fungus; Soup with tomatoes, bean curd skin and king oyster mushroom; Pineapple. Dinner (**Right**): Brown rice noodle with fried bean paste-flavored hard tofu cubes, ground pork, chicken cubes, and vegetables; Winter squash and clam soup; Pear. Recipes and nutrient data analysis are available in the Supplementary Materials.

**Figure 3:**
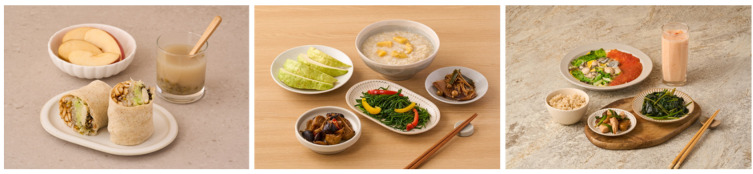
**Suggested plant-based diet plan B at 2100 Kcal level.** (**Left**) Breakfast: Taiwanese spring roll made with whole wheat wrap containing tofu shreds, mung bean sprouts, cabbage shreds, lima bean, raisin, peanut powder, and sesame powder; Apple; Morning drink made with mung bean & Job’s tear. (**Center**) Lunch: Sweet potato and brown rice congee; Stewed milkfish; Braised mushroom and wheat gluten rolls; Stir-fried bell pepper and white water snowflake stems; Guava. (**Right**) Dinner: Steamed brown rice; Pan-fried oyster and egg with green vegetable and minced tomato; Braised chicken with chestnut; Boiled sweet potato leaves; Papaya and Unsweetened yogurt. Recipes and nutrient data analysis are available in the Supplementary Materials.

Some of these ideas have been used in designing an EAT-*Lancet* two-day menu (see Figure 2 and Figure 3). From these menus, the pan-fried oyster egg with green vegetable and topped with tomato sauce was selected, since this dish is a local delicacy and the zinc- and B vitamin-rich oyster is considered environmentally friendly. A famous noodle sauce, Ja-Chiang, is made by stir-frying hard tofu cubes and ground pork with fried fermented bean paste. Instead, we replaced half (or it can be more than half) of the ground pork with diced chicken without affecting the taste. The nutrition values of these dishes are also provided in the appendices.

### 4.4. Implications and Proposal for the Future Taiwanese EAT-Lancet Guide and Food Innovations

Compared to the Taiwanese Daily Food Guide and the EAT-*Lancet* guidelines, the Taiwanese low red meat dietary pattern is not altogether satisfactory. It is crucial to be in line with the Taiwan Food Guide. For example, the mean vegetable (2.6 servings a day) and fruit (1.7~1.8 servings a day) consumption levels have not reached the five-a-day recommendation, not to mention the seven servings recommended to those consuming higher level of calories. Furthermore, the intake of added sugar is near 8–10% of total caloric consumption, which may be lowered to 3% by substituting UPFs with whole/healthy foods. Instead of preventing people from eating any sweets, creating sweets with whole fruits may be a desirable alternative so as to increase fruit consumption at the same time as lower the consumption of added sugar. 

Mean dairy consumption has been at the level of half a serving a day for the past 3 decades for average Taiwanese men and women [16]. This departs greatly from the recommendation of 1 to 2 servings a day, which appears unrealistic considering the lactose intolerance problem in the Taiwanese population (prevalence rate: 88%) [17]. Unlike in other countries, lactose-free products are not available. Naturally fermented dairy products with low lactose content are consumed by just a fraction of the population. Therefore, dairy consumption remains very low due to these cultural and food-environmental barriers [18]. However, some, but not too much, dairy should be promoted particularly for Asians, given its beneficial effects on all-cause and cardiovascular mortality, over-fatness (by BMI and abdominal circumference) and diabetes, as well as on fractures and their sequelae [18,19]. Therefore, we would advocate reducing dairy recommendations to at most 1 serving a day according to the EAT-*Lancet* guidelines. Simultaneously, we encourage the promotion of vitamin D, calcium, and preferably certain B vitamins and magnesium-enriched plant-based protein drinks that mimic the nutrient profile of milk. Soymilk has historically been the most preferred morning protein drink in Taiwan, and its fortification would make nutrition education and consultation easier with respect to protein-rich morning beverages. Regarding the concern over the potential hazards of calcium insufficiency from the reduced 0.5 portions of dairy, a serving of milk or fortified soymilk combined with a certain amount of dark green leafy vegetables, as promoted by the Taiwanese Food Guide and the EAT-*Lancet* principles, may ensure proper calcium intake. Our proposed local PHD also demonstrated that consuming one-third to one-half of the recommended vegetables from the dark green leafy vegetables category could help to raise calcium content in the diet. In order to meet the original protein recommendation in the Daily Food Guide, we suggest adding the half a serving to the non-dairy protein food group. 

With respect to the non-dairy protein foods consumed in Taiwan, the amount of beef or lamb consumption is currently very low. Instead, pork has been the major protein food in Taiwan for a long time, so it is a challenge to lower pork intakes. However, the pork in certain lean pork dishes using ground, sliced, or shredded pork may be replaced by similarly-cut forms of chicken meat (white or dark), aquatic foods, and firmed soybean curd (in slices, threads, or cubes) without affecting tastes. Diverse, traditional tofu dishes may also be revitalized. The self-sufficiency rate for aquatic foods is as high as 132% in Taiwan [20]. Consumption of aquatic foods is a healthy alternative to pork and there are versatile ways to cook seafoods. In addition, low-temperature-roasted, non-salted nuts and tree nuts may also be considered as protein sources and used in main dishes, since their tastes are gaining popularity and there are many popular Chinese or Taiwanese dishes made from mixing peanuts and cashews, etc., with other protein foods. More may be developed in the near future. We have proposed our serving numbers with respect to these protein foods in Table 5, considering EAT-*Lancet* principles.

Although mean consumption levels for other food groups such as starchy staple foods and cooking oils are mostly right on the mark with respect to the Taiwan Food Guide, the key problem for grains and starchy foods is that people customarily eat white rice- and white flour-made products, which not only jeopardizes the nutrient status of dietary fiber and minerals such as magnesium, calcium, and zinc, but may also in part contribute to the high prevalence of diabetes in Taiwan [21,22]. Clearly, beyond educating the public to substitute one-third or more of the white rice consumed with whole grains, efforts such as innovative reformulation for pastries and desserts are needed to lower refined sugar and to increase the proportion of whole grains and starchy roots and beans in the staple category. 

### 4.5. Limitations

The present study had some limitations. Firstly, the dietary data were obtained using the 24-h dietary recall method, which estimates nutrient intake based on nutrient values of foods in the food composition table. The Taiwanese food composition table provides nutrient data for most raw foods and some processed and packaged foods. Nutrient lost during storage and cooking processes in the restaurant or at home has not been accounted for. Secondly, data on added sugars are only partially available in our local food composition table. The missing data have been imputed using added sugar content estimations provided by a local publication [23].

## 5. Conclusions

When adopting EAT-Lancet principles to promote sustainability, it is important to carry out local empirical investigations to explore whether these principles are indeed beneficial in most aspects of health across different regions of the world. This study primarily focused on nutrient adequacy as an example of the local translation process. We have considered representative local dietary components and ingredients under the EAT-Lancet guidelines. We have also tried to modify local dishes to ensure their acceptance. Most importantly, we have assessed whether reducing red meat and increasing plant-based foods can meet the nutritional needs of people. 

From this evidence-based exercise, we discovered that the Taiwanese low red meat and low UPFs dietary pattern provides a better nutrient profile compared to a diet high in red meat and UPFs. If this local pattern is further refined according to the Taiwanese Food Guide and EAT-Lancet principles, it could achieve near-perfect nutrient composition and improve many health-related dietary aspects of the populace, such as fiber content, added sugar percentage, and fatty acids profiles. We did find that zinc levels were slightly compromised, and have suggested promoting certain local, eco-friendly, zinc-rich foods as a remedy. It is crucial to continuously monitor the nutritional status of the population and to adjust public health nutrition policies whenever necessary. 

## Figures and Tables

**Table 1:** Descriptive characteristics by levels of red meat consumption in men and women, Nutrition and Health Survey in Taiwan 2017–2020 ^1^.

	*n*	Low	Medium	High	*P* for Trend ^2^
*Male*					
Red meat (Kcal)	867/1749/619	25.0 ± 2.2	276.0 ± 6.1	743.4 ± 21.7	<0.0001
Age (years)	867/1749/619	49.7 ± 0.9	45.3 ± 0.5	45.0 ± 0.9	0.002
BMI (kg/m^2^)	618/1212/419	24.9 ± 0.3	25.3 ± 0.2	25.7 ± 0.4	0.175
Educational level					0.036
Below junior school	223/322/115	11.6	6.8	7.0	
Junior and high school	364/712/250	40.4	39.9	36.7	
College or above	280/715/254	48.1	53.4	56.3	
Marital status					0.901
Unmarried	153/396/152	31.3	34.0	32.4	
Married	598/1146/384	59.3	57.4	57.5	
Divorced, separated, or widowed	116/207/83	9.4	6.7	10.1	
Cigarette use					0.037
Never	370/691/198	50.0	46.5	42.1	
Quit	299/527/188	24.3	22.4	20.5	
Current	188/520/224	25.7	31.2	37.4	
Alcohol use					0.055
Never	206/387/101	28.2	22.5	15.6	
Quit	285/717/287	44.4	48.7	56.3	
Current	175/377/137	27.4	28.8	28.1	
*Female*					
Red meat (Kcal)	1229/1592/482	20.7 ± 1.15	200.2 ± 4.7	578.5 ± 32.2	<0.0001
Age (years)	1229/1592/482	50.3 ± 0.7	46.7 ± 0.5	43.2 ± 1.0	0.0001
BMI (kg/m^2^)	808/1120/325	23.8 ± 0.2	23.7 ± 0.2	23.8 ± 0.4	0.823
Educational level					0.002
Below junior school	522/501/122	21.9	16.0	10.7	
Junior and high school	398/574/190	35.9	36.0	37.1	
College or above	309/516/169	42.1	48.0	52.2	
Marital status					0.014
Unmarried	181/266/96	21.6	24.6	27.8	
Married	690/978/279	58.5	62.5	61.1	
Divorced, separated, or widowed	358/346/106	19.8	12.9	11.1	
Cigarette use					0.372
Never	1107/1439/408	89.0	90.5	85.8	
Quit	48/50/22	5.8	4.5	4.8	
Current	52/86/47	5.2	5.0	9.4	
Alcohol use					0.175
Never	689/774/199	46.6	42.8	37.2	
Quit	224/338/143	27.6	27.6	37.7	
Current	229/366/106	25.9	29.6	25.1	

Abbreviations: BMI, body mass index. ^1^ Values are presented as *n*, mean ± SEM, or %. ^2^ Within each sex group, a trend test was performed with a generalized linear model for continuous variables or a chi-square test for categorical variables to examine whether the variables had an ordered relationship with levels of red meat. Data of red meat were cube root transformed and that of BMI were log transformed for trend tests.

**Table 2:** Mean uncooked food weights across three red meat groups by sex in Taiwanese adults: Nutrition and Health Survey in Taiwan 2017–2020 ^1^.

	Male				Female			
	Low	Medium	High	*P* for Trend ^2^	Low	Medium	High	*P* for Trend ^2^
Grains and roots								
Rice, wheat, and other grains	258.5 ± 13	278.7 ± 4.9	230.5 ± 8.7	0.308	206.1 ± 5.5	194.2 ± 5.4	147.8 ± 6.3	<0.0001
Potatoes, cassava, corn, and other roots	29.7 ± 3.7	35.2 ± 3.1	21.8 ± 2.7	0.987	30.7 ± 2.4	34.0 ± 2.8	33.4 ± 5.1	0.997
Dry beans, lentils, and peas	9.1 ± 5.7	8.1 ± 2.1	2.8 ± 0.7	0.789	3.1 ± 0.6	5.7 ± 0.9	7.1 ± 3.6	0.781
Protein foods								
Beef and lamb	0.7 ± 0.3	19.3 ± 1.8	62.0 ± 7.4	<0.0001	0.6 ± 0.2	12.7 ± 2.1	55.9 ± 19.1	<0.001
Pork	15.1 ± 1.0	109.1 ± 3.0	249.5 ± 10	<0.0001	13.5 ± 1.0	82.6 ± 2.6	180.4 ± 10.4	<0.0001
Poultry	75.4 ± 7.0	69.3 ± 4.4	44.0 ± 5.6	0.014	52.7 ± 4.0	43.7 ± 3.1	25.6 ± 3.7	<0.0001
Egg	42.1 ± 3.2	49.2 ± 2.2	40.6 ± 3.8	0.500	38.9 ± 3.0	40.9 ± 1.7	36.6 ± 2.8	0.814
Fish and Seafood	59.3 ± 6.1	58.2 ± 3.7	55.3 ± 6.1	0.806	41.0 ± 2.3	52.9 ± 3.3	31.8 ± 3.5	0.141
Soy	26.0 ± 3.6	24.6 ± 2.2	19.5 ± 2.1	0.452	31.2 ± 4.4	16.7 ± 1.5	13.8 ± 1.7	0.005
Peanut	3.8 ± 1.1	2.2 ± 0.3	2.0 ± 0.7	0.380	1.9 ± 0.4	1.7 ± 0.3	1.3 ± 0.5	0.629
Nut	4.3 ± 0.8	2.2 ± 0.3	4.5 ± 2.0	0.330	5.2 ± 0.7	3.0 ± 0.4	1.5 ± 0.6	0.014
Dairy foods	100.2 ± 12	132.3 ± 9.9	120.0 ± 14.8	0.213	126.8 ± 10.8	148 ± 13.3	112.2 ± 16.7	0.175
Vegetable								
Dark green	93.5 ± 7.4	85.2 ± 3.9	86.6 ± 7.3	0.096	88.0 ± 5.8	96.3 ± 5.0	81.6 ± 7.0	0.300
Red and orange	12.2 ± 1.5	9.5 ± 0.9	7.4 ± 1.4	0.116	9.3 ± 1.0	9.9 ± 1.1	12.3 ± 2.0	0.482
Other	155.7 ± 9.3	152.6 ± 4.3	171.2 ± 11.7	0.016	158.7 ± 8.7	150.5 ± 6.1	159.6 ± 13.2	0.456
Fruit	176.3 ± 12.8	166.7 ± 8.9	138.9 ± 15.7	0.159	191.7 ± 10.8	185.7 ± 8.9	133.1 ± 12.7	0.008
Added sugar	47.6 ± 3.8	56.9 ± 2.5	48.3 ± 3.7	0.973	38.3 ± 2.3	39.4 ± 1.8	37.4 ± 3.9	0.487
Added oils and fats								
Palm	1.4 ± 0.2	2.5 ± 0.2	1.5 ± 0.2	0.100	1.1 ± 0.1	1.5 ± 0.2	1.0 ± 0.2	0.678
Vegetable	13.9 ± 0.9	24.0 ± 1.0	20.5 ± 1.2	0.001	13.1 ± 0.8	17.6 ± 0.6	16.1 ± 1.4	0.415
Animal	12.4 ± 1.3	12.5 ± 0.7	8.2 ± 0.7	0.049	11.7 ± 0.8	10.3 ± 0.5	9.0 ± 1.0	0.268

^1^ All values are presented as mean ± SEM in grams. Sample sizes for the low/medium/high groups in males and females were 867/1749/619 and 1229/1592/482, respectively. Alcohol, salt, soy sauce, soy sauce paste, and Chinese herbs represent none of the six food groups, and their contribution to total energy is low, so they are not presented in this table. ^2^ Data were cube root-transformed for trend tests. Trend tests were performed using a generalized linear model with age adjustment to examine whether the intakes of red meat had an ordered relationship with raw food weights of the six food groups.

**Table 3:** Total energy intakes and energy contribution from macronutrients, SFAs, and added sugar across three levels of red meat intake groups before and after replacing UPFs with whole/healthy foods, by sex, in Taiwanese adults: Nutrition and Health Survey in Taiwan 2017–2020 ^1^.

	Before ^2^				After ^2^			
	Low	Medium	High	*P* for Trend ^3^	Low	Medium	High	*P* for Trend ^3^
*Male*								
Energy (Kcal)	1984 ± 64	2433 ± 40	2607 ± 73	<0.0001	1814 ± 58	2143 ± 31	2158 ± 62	0.002
Carbohydrate (%)	55.9 ± 0.5	51.2 ± 0.4	42.7 ± 0.6	<0.0001	53.9 ± 0.6	50.6 ± 0.4	45.1 ± 0.6	<0.0001
Protein (%)	16.0 ± 0.3	16.8 ± 0.2	19.1 ± 0.3	<0.0001	18.0 ± 0.3	19.4 ± 0.2	23.5 ± 0.3	<0.0001
Fat (%)	28.1 ± 0.5	32.0 ± 0.3	38.2 ± 0.5	<0.0001	28.2 ± 0.5	30.1 ± 0.3	31.5 ± 0.5	0.001
SFAs (%)	8.6 ± 0.2	10.5 ± 0.1	13.3 ± 0.2	<0.0001	7.9 ± 0.2	9.1 ± 0.1	9.9 ± 0.2	<0.0001
Added sugar (%)	7.6 ± 0.6	7.4 ± 0.2	6.1 ± 0.5	0.051	3.2 ± 0.3	3.2 ± 0.1	2.7 ± 0.2	0.757
*Female*								
Energy (Kcal)	1654 ± 38	1797 ± 27	1858 ± 55	0.013	1533 ± 40	1606 ± 24	1489 ± 36	0.194
Carbohydrate (%)	54.3 ± 0.6	50.0 ± 0.3	41.4 ± 0.6	<0.0001	52.6 ± 0.5	49.3 ± 0.4	42.6 ± 0.8	<0.0001
Protein (%)	15.8 ± 0.3	17.4 ± 0.2	19.4 ± 0.4	<0.0001	17.4 ± 0.3	19.8 ± 0.2	24.4 ± 0.5	<0.0001
Fat (%)	29.9 ± 0.4	32.7 ± 0.3	39.2 ± 0.6	<0.0001	30.1 ± 0.4	30.9 ± 0.3	33.0 ± 0.6	0.005
SFAs (%)	8.9 ± 0.2	10.9 ± 0.2	14.1 ± 0.3	<0.0001	8.3 ± 0.2	9.5 ± 0.2	10.9 ± 0.2	<0.0001
Added sugar (%)	6.9 ± 0.5	6.4 ± 0.3	6.6 ± 0.6	0.225	3.1 ± 0.2	3.3 ± 0.1	3.1 ± 0.3	0.975

Abbreviation: SFAs, saturated fatty acids. ^1^ All values are presented as mean ± SEM. Sample sizes for low/medium/high groups in males and females were 867/1749/619 and 1229/1592/482, respectively. ^2^ “Before” represents the actual intake of the original diet, and “After” means all the ultra-processed foods of the original diet were substituted with whole foods. ^3^ Data were cube root-transformed for trend tests. Trend tests were performed using a generalized linear model with age adjustment to examine whether the intake of red meat had an ordered relationship with total energy intake, energy contribution from macronutrients, SFAs, and added sugar.

**Table 4:** Average nutrient adequacy ratio (as % of DRIs) and PMS ratio across three levels of red meat intake groups before and after replacing UPFs with whole/healthy foods, by sex, in Taiwanese adults: Nutrition and Health Survey in Taiwan 2017–2020 ^1^.

	Before ^2^				After ^2^			
	Low	Medium	High	*P* for Trend ^3^	Low	Medium	High	*P* for Trend ^3^
*Male*								
Protein	117 ± 2	124 ± 1	139 ± 2	<0.0001	131 ± 2	143 ± 2	169 ± 3	<0.0001
Vitamin B1	91 ± 1	120 ± 1	155 ± 6	<0.0001	115 ± 1	174 ± 2	261 ± 8	<0.0001
Vitamin B2	102 ± 3	102 ± 2	108 ± 4	0.084	112 ± 3	116 ± 2	131 ± 4	<0.001
Niacin	114 ± 4	119 ± 2	137 ± 3	<0.0001	137 ± 4	147 ± 2	184 ± 4	<0.0001
Vitamin B6	139 ± 4	136 ± 3	146 ± 4	0.034	158 ± 5	169 ± 3	208 ± 5	<0.0001
Vitamin B12	214 ± 25	212 ± 9	258 ± 20	<0.0001	231 ± 32	225 ± 9	298 ± 22	<0.0001
Vitamin A	162 ± 10	117 ± 4	127 ± 13	0.054	170 ± 10	127 ± 4	145 ± 14	0.131
Vitamin C	179 ± 12	148 ± 6	127 ± 8	0.015	204 ± 14	171 ± 6	157 ± 10	0.024
Vitamin D	74 ± 6	55 ± 2	53 ± 4	0.585	74 ± 6	52 ± 2	51 ± 5	0.219
Vitamin E	84 ± 3	74 ± 1	66 ± 2	<0.001	97 ± 3	92 ± 1	84 ± 2	0.003
Calcium	59 ± 3	51 ± 2	44 ± 2	<0.001	63 ± 3	56 ± 2	51 ± 2	0.005
Phosphorus	155 ± 2	154 ± 2	155 ± 3	0.526	183 ± 2	182 ± 2	192 ± 3	0.009
Iron	151 ± 4	154 ± 3	143 ± 4	0.317	167 ± 4	173 ± 4	173 ± 4	0.162
Zinc	74 ± 2	81 ± 1	99 ± 3	<0.0001	84 ± 2	92 ± 1	108 ± 3	<0.0001
Magnesium	86 ± 2	74 ± 1	69 ± 1	<0.0001	109 ± 2	98 ± 1	97 ± 2	0.001
Potassium	100 ± 2	90 ± 1	92 ± 2	0.329	114 ± 2	108 ± 1	120 ± 3	0.008
Sodium	135 ± 4	151 ± 2	149 ± 4	0.032	130 ± 6	140 ± 2	146 ± 4	0.024
Dietary fiber	63 ± 2	52 ± 1	43 ± 1	<0.0001	74 ± 2	63 ± 1	56 ± 2	<0.0001
P/S ratio	1.25 ± 0.05	1 ± 0.02	0.75 ± 0.01	<0.0001	1.41 ± 0.05	1.21 ± 0.02	1.00 ± 0.02	<0.0001
M/S ratio	1.25 ± 0.03	1.15 ± 0.01	1.14 ± 0.01	0.208	1.34 ± 0.03	1.22 ± 0.01	1.22 ± 0.02	0.084
*Female*								
Protein	109 ± 2	120 ± 1	134 ± 2	<0.0001	120 ± 2	136 ± 1	166 ± 3	<0.0001
Vitamin B1	103 ± 2	137 ± 2	178 ± 7	<0.0001	124 ± 2	188 ± 2	298 ± 10	<0.0001
Vitamin B2	115 ± 2	122 ± 2	121 ± 3	0.034	126 ± 3	136 ± 2	149 ± 3	<0.0001
Niacin	105 ± 4	113 ± 2	125 ± 3	<0.0001	124 ± 4	134 ± 2	168 ± 4	<0.0001
Vitamin B6	118 ± 4	113 ± 2	118 ± 3	0.094	132 ± 4	137 ± 2	172 ± 5	<0.0001
Vitamin B12	151 ± 12	203 ± 13	207 ± 11	<0.0001	154 ± 13	218 ± 15	244 ± 13	<0.0001
Vitamin A	164 ± 8	160 ± 7	132 ± 8	0.253	174 ± 9	173 ± 8	153 ± 9	0.898
Vitamin C	175 ± 8	155 ± 8	124 ± 10	0.006	195 ± 9	175 ± 8	152 ± 11	0.039
Vitamin D	58 ± 4	54 ± 2	38 ± 3	0.094	57 ± 4	52 ± 3	36 ± 3	0.043
Vitamin E	75 ± 2	68 ± 1	59 ± 3	<0.0001	87 ± 1	82 ± 1	74 ± 3	0.001
Calcium	54 ± 2	49 ± 1	41 ± 2	<0.001	59 ± 2	53 ± 1	48 ± 2	0.007
Phosphorus	127 ± 2	130 ± 1	131 ± 2	0.037	147 ± 2	150 ± 1	162 ± 2	<0.0001
Iron	90 ± 2	88 ± 2	83 ± 2	0.330	98 ± 2	97 ± 2	100 ± 2	0.033
Zinc	75 ± 1	85 ± 1	102 ± 4	<0.0001	84 ± 1	95 ± 1	114 ± 5	<0.0001
Magnesium	87 ± 2	79 ± 1	70 ± 2	<0.001	107 ± 2	100 ± 1	96 ± 2	0.028
Potassium	96 ± 1	96 ± 2	95 ± 2	0.092	109 ± 2	112 ± 2	123 ± 2	<0.0001
Sodium	109 ± 3	124 ± 2	131 ± 6	0.003	105 ± 3	115 ± 2	123 ± 6	0.012
Dietary fiber	75 ± 2	67 ± 1	54 ± 2	<0.001	87 ± 2	79 ± 2	68 ± 2	<0.001
P/S ratio	1.29 ± 0.04	0.96 ± 0.02	0.71 ± 0.02	<0.0001	1.45 ± 0.04	1.15 ± 0.03	0.92 ± 0.03	<0.0001
M/S ratio	1.25 ± 0.02	1.14 ± 0.01	1.13 ± 0.01	0.063	1.33 ± 0.02	1.22 ± 0.01	1.19 ± 0.02	0.033

Abbreviations: DRIs, Dietary Reference Intakes; M, monounsaturated fatty acids; P, polyunsaturated fatty acids; S, saturated fatty acids; UPFs, ultra-processed foods. ^1^ All values are presented as mean ± SEM. Sample sizes for low/medium/high groups in males and females were 867/1749/619 and 1229/1592/482, respectively. Every male and female participant’s daily total dietary intake was firstly adjusted to 2100 Kcal and 1650 Kcal, respectively, and then compared to the standards for 31~50-year-old males and females with slightly low physical activity levels according to the eighth edition of Taiwanese DRIs. ^2^ “Before” represents the actual intake of the original diet, and “After” means all the UPFs of the original diet were substituted with whole/healthy foods. ^3^ Data were cube root-transformed for trend tests. Trend tests were performed using a generalized linear model with age adjustment to examine whether the intakes of red meat had an ordered relationship with protein intakes, multiple various micronutrient intakes, PMS ratios, and dietary fiber intakes.

**Table 5:** Dietary structure of the “Taiwanese diet with low red meat and low UPFs”, the “Taiwanese Daily Food Guide”, and the “proposed local planetary health diet”.

	Low Red Meat with Low UPFs Diet in Male	Taiwanese Daily Food Guide	Proposed Local Planetary Health Diet	Low Red Meat with Low UPFs Diet in Female	Taiwanese Daily Food Guide	Proposed Local Planetary Health Diet
**Energy intake (Kcal)**	**1814**	**2100**	**2100**	**1533**	**1600~1700**	**1650**
**Grains and roots (serving number)**	**11.4**	**12**	**12**	**8.8**	**10**	**10**
Rice, wheat, and other grains	10.7		11	8.1		9
Potatoes, cassava, corn, and other roots	0.5		0.5	0.6		0.5
Dry beans, lentils, and peas	0.2		0.5	0.1		0.5
**Fruit & added sugar (serving number)**	**1.7 & 0.9**	**3.5**	**3.5 & 1**	**1.8 & 0.7**	**2**	**2 & 1**
Fruit	1.7	3.5	3.5	1.8	2	2
Added sugar	0.9		1	0.7		1
**Vegetable (serving number)**	**2.6**	**4**	**4**	**2.6**	**3**	**3**
Dark green	0.8		2	0.7		1
Red and orange	0.2		0.5	0.1		0.5
Other	1.7		1.5	1.7		1.5
**Dairy foods (serving number)**	**0.4**	1.5 (−0.5 *)	**1**	**0.5**	1.5 (−0.5 *)	**1**
**Protein sources & nuts/seeds (serving number)**	**6.1 & 0.5**	**6** (+0.5 *) & (+0.5 **)	**6.5 & 0.5**	**5.0 & 0.4**	**4.5** (+0.5 *) & (+0.5 **)	**5 & 0.5**
Beef and lamb	0.02		Small amt	0.02		Small amt
Pork	0.4		0.5	0.3		0.5
Poultry	2.1		1.0	1.4		0.5
Egg	0.7		0.5	0.7		0.5
Fish and seafood	1.6		2.0	1.1		1.5
Soy	1.3		2.5	1.6		2.0
Nuts/peanut	0.48	(+0.5 **)	0.5	0.36	(+0.5 **)	0.5
**Added fats (serving number)**	**4**	5 (−1 **)	**4**	**4.6**	3~4 (−1 **)	**2** **~3**
Animal fats	1.7		0	1.9		0
Palm oil	0.04		Small amt	0.2		Small amt
Vegetable oils	2.3	4	4	2.5	2**~**3	2**~**3
Nuts		1 (−1 **)			1 (−1 **)	

Abbreviations: amt, amount; UPFs, ultra-processed foods. *: A half serving of dairy foods is close to a half serving of protein sources. A half serving of dairy from the Taiwan Food Guide structure is reallocated to the non-dairy protein food group in EAT-*Lancet* dietary structure. **: Nuts are included in the oils/fats group in the Taiwanese Daily Food Guide. One serving of nuts in the oils/fats group (with 5 g fat/oil) becomes half a serving of the non-dairy protein foods group (with 4 g of protein). Nuts are reallocated to the non-dairy protein foods group in the EAT-*Lancet* dietary structure. (): Parenthesis include no. of servings for which we suggest reallocation.

**Table 6:** Energy distribution for macronutrients, nutrient adequacy (as % of DRIs), and PMS ratio by sex: Taiwanese diet with low red meat before and after UPFs substitution, and proposed local planetary health diet ^1^.

	Male			Female		
	Before ^2^	After ^2^	Proposed Local Planetary Health Diet ^2^	Before ^2^	After ^2^	Proposed Local Planetary Health Diet ^2^
Energy (Kcal)	1984	1814	2155	1654	1533	1658
Carbohydrate (%)	56	54	56	54	53	56
Protein (%)	16	18	17	16	17	18
Fat (%)	28	28	26	30	30	27
SFA (%)	8.6	7.9	6.8	8.9	8.3	7.4
Added sugar (%)	9.9	3.2	2.7	8.9	3.1	3.5
Nutrient adequacy ratio (% DRIs)						
Protein	117	131	130	109	120	122
Vitamin B1	91	115	131	103	124	141
Vitamin B2	102	112	122	115	126	126
Niacin	114	137	126	105	124	118
Vitamin B6	139	158	180	118	132	135
Vitamin B12	214	231	218	151	154	187
Vitamin A	162	170	291	164	174	233
Vitamin C	179	204	227	175	195	163
Vitamin D	74	74	73	58	57	64
Vitamin E	84	97	106	75	87	77
Calcium	59	63	91	54	59	72
Phosphorus	155	183	195	127	147	155
Iron	151	167	197	90	98	102
Zinc	74	84	90	75	84	90
Magnesium	86	109	130	87	107	116
Potassium	100	114	139	96	109	112
Sodium	135	130	123	109	105	96
Dietary fiber	63	74	95	75	87	95
PMS ratio						
P/S ratio	1.25	1.41	1.55	1.29	1.45	1.36
M/S ratio	1.25	1.34	1.30	1.25	1.33	1.20

Abbreviations: DRIs, Dietary Reference Intakes; M, monounsaturated fatty acids; P, polyunsaturated fatty acids; S, saturated fatty acids; UPFs, ultra-processed foods. ^1^ All values are presented as mean percentages. Sample sizes were 867 and 1229 for males and females, respectively. Every male and female participant’s daily total dietary intake was firstly adjusted to 2100 Kcal and 1650 Kcal, and then compared to the standards of the eighth edition of Taiwanese DRIs for 31~50-year-old males and females at slightly low physical activity level. ^2^ “Before” represents the actual intake of the low red meat group, “After” means most of the UPFs in the original diet were substituted with whole/healthy foods, and “Proposed local planetary health diet” indicates the dietary structure of the low red meat group was adjusted toward both the Taiwanese Food Guide and the EAT-*Lancet* principles.

## Data Availability

The datasets used in the current study are not publicly available due to the legal restrictions of the Personal Information Protection Act legislated by the government of Taiwan. Data of Nutrition and Health Survey from Taiwan described in the manuscript belong to HPA, which can be accessed in the data center with the permission of HPA.

## Supplementary Materials

The following supporting information can be downloaded at https://www.mdpi.com/article/10.3390/nu16162775/s1: Table S1: Food substitution lists; Table S2: Definition of one serving for the six food groups in Taiwanese Daily Food Guide; Table S3: Nutrient analysis for the suggested plant-based diet plan A at 2100 Kcal level ^a.^; Table S4: Nutrient analysis for the suggested plant-based diet plan B at 2100 Kcal level ^a.^; Figure S1: Recipes for “Clam and winter melon soup, flavored with ginger”; Figure S2: Recipes for “Brown rice spaghetti with “stirred fried and fermented soybean paste flavored” hard bean curd cube, chicken cube, and ground pork”; Figure S3: Recipes for “Braised milk fish with ginger and scallion”.

## Abbreviations

DRIs: Daily Reference Intakes, M/S ratio: monounsaturated to saturated fatty acids ratio, NAHSIT: Nutrition and Health Survey in Taiwan, PBD: plant-based diet; PHD, planetary health diet; P/S ratio: polyunsaturated to saturated fatty acids ratio, PMS ratios: polyunsaturated and monounsaturated to saturated fatty acids ratios, SFAs: saturated fatty acids, UPFs: ultra-processed foods.
