# Supplementary material for: Is Nutrient Quality of the Locally-Existing, EAT-Lancet-like Plant-Based Diet Better or Worse than the Average Diet in Taiwan? An Example of Local Translation
Source: Nutrients. 2024 Aug 20;16(16):2775. doi: 10.3390/nu16162775 (PMC11356800; doi:10.3390/nu16162775)
Supplement: Supplementary file 1 [file nutrients-16-02775-s001.zip › nutrients-3085702-supplementary.pdf]

nutrients

MDPI

Supplementary Materials

Is Nutrient Quality of the Locally Existing Eat-*Lancet* Like Plant-Based Diet Better or Worse than the Average Diet in Taiwan? An Example of Local Translation

Wen-Harn Pan [1-5,†,*], Szu-Yun Wu [1,†] and Po-Chen Chang [2,5]

Table S1. Food substitution lists.

Consumed foods	Substitutes
Refined rice and rice products, e.g., rice cake, rice noodles, sticky rice balls, and mochi	One third by brown rice
Refined wheat and flour products, e.g., white noodle, yellow noodles, steamed bun, dumpling wrapper, (flaky) scallion pancake, clay oven rolls, and white flour	One thirds by buckwheat noodle, whole grain breads, or whole grain flour
Refined white bread, white toast and biscuit snacks	Whole grain breads
Refined tubers or starchy vegetables, e.g., fries, potato starch, taro balls	One third by original foods
Refined dried seeds, other grains, and their products, e.g., mung bean starch, sweet adzuki bean paste, popcorns, and sweet barley milk	One third by original foods
Margarine and shortening used in fried noodles, fried eggs, and thickened soups	Locally representative vegetable oils
Animal cooking oils	Locally representative vegetable oils
Fried, salted, and candied nuts	Unflavored lightly baked ones
The spreads such as butter, garlic flavored fats, shortening, and soufflé	Locally representative mixed nuts
High fat meats	Medium fat meats
Fried chicken	Roast chicken
Processed meatball, fish ball, and dumplings with seafood fillings made for hot pot	Home-made meatball and aquatic foods
Small amount of animal skin	Deleted
Processed egg products	Fresh eggs
Whole milk, condensed milk, and ice cream	Low-fat milk

Processed soybean products	Locally representative tofu and wheat gluten rolls
Fried tofu and fried bean curd skin	Fresh hard tofu
Sugar-containing products	Sugar-free products
Pickled vegetables	Fresh vegetables
Fried vegetable products, e.g., fried mushroom	Dried mushroom
Candied fruit and sugary jams	Fresh fruits
Sugar-contained dried fruits	Raisins

Table S2. Definition of one serving for the six food groups in Taiwanese Daily Food Guide.

	Protein (g)	Fat (g)	Carbohydrate (g)
**Grains and roots**			
Rice, wheat, and other grains/ Potatoes, cassava, corn, and other roots/ Dry beans, lentils, and peas	2	+	15*
**Protein foods**			
Low fat	7*	3	+
Medium fat	7*	5	+
High fat	7*	10	+
Peanuts/ Tree nuts	8	10*	
**Dairy foods**			
Whole fat	8*	8	12
Low fat	8*	4	12
No fat	8*	+	12
**Vegetables**	1*	+	5*
**Fruits**	+	+	15*
**Added fats and oils**		5*	

+: Trace amount.

*: Denote the macronutrient(s) used to estimate the serving size for that specific food group. When two macronutrients are marked, they are weighted by their caloric values.

Table S3. Nutrient analysis for the suggested plant-based diet plan A at 2100 Kcal level.[a.]

	Breakfast			Lunch				Dinner				
	Steamed whole wheat buns with a slice of cheese and a pan-fried egg	Dragon Fruit (red color)	Sesame and honey-flavored morning drink with half low-fat milk and half soy bean milk	Steamed black rice with chick-peas	Steamed squid	Soup with tomatoes, bean curd skin and king oyster mushroom	Stir-fried water spinach and wood ear	Pineapple	Taiwanese Jajang brown rice noodle	Winter squash and clam soup	Pear	Total	DRIs (%)
Energy (kcal)	438.58	58.96	214.6	294.45	47.97	99.6	53.13	61.32	527.28	74.34	58.26	1928.5	91.8
Carbohydrate (g)	51.94	14.46	24.87	59.33	0.72	6.44	6.04	15.91	68.61	5.97	15.58	269.87	
Dietary fiber (g)	3.35	1.47	2.43	11.78	0.05	1.69	3.73	1.17	5.96	1.7	1.51	34.86	120.2
Protein (g)	18.99	1.59	8.81	7.67	5.62	8.74	2.13	0.85	23.09	8.29	0.39	86.18	123.1
Fat (g)	17.55	0.19	9.63	2.48	2.64	5.29	2.73	0.14	18.18	3.09	0.1	62.03	
SFA (g)	7.13	0	2.78	0.6	0.44	0.83	0.5	0.04	3.36	0.59	0.04	16.32	
MUFA (g)	5.47	0	3.13	1	0.81	1.42	0.6	0.08	5.03	0.94	0.04	18.51	
PUFA (g)	4.94	0	3.72	0.87	1.4	3.04	1.62	0.02	9.7	1.51	0.03	26.84	
Cholesterol (mg)	223.6	0	12	0	110.57	0	0	0	25.61	39.32	0	411.11	
Vitamin A (μg RE)	151.43	0	23.6	1.36	5.25	99.18	182.18	3.37	322.69	13.07	0	802.13	133.7
Vitamin C (mg)	5.86	5.77	0.59	7.18	0.08	9.39	10.74	9.41	12.06	21.36	2.92	85.37	85.4
Vitamin D (μg)	1.04	0	0.03	0	0	0.3	3.41	0	0.03	0	0	4.8	48
Vitamin E (mg α-TE)	2.42	0.27	0.4	0.94	0.64	0.87	0.84	0.02	2.77	0.47	0.06	9.7	80.8
Vitamin B1 (mg)	0.18	0.04	0.16	0.32	0.02	0.09	0.05	0.09	0.56	0.08	0.04	1.62	135.2
Vitamin B2 (mg)	0.51	0.04	0.26	0.08	0.02	0.08	0.14	0.05	0.21	0.82	0.03	2.23	171.2
Niacin (mg)	1.72	0	0.9	4.78	1.34	2.07	0.77	0.21	4.55	1.5	0.57	18.42	115.1
Vitamin B6 (mg)	0.19	0.01	0.11	0.21	0.01	0.29	0.16	0.17	0.48	0.08	0.02	1.74	115.8
Vitamin B12 (μg)	0.87	0	0.15	0	1.48	0.02	0	0	0.13	50.54	0	53.19	2216.1
Calcium (mg)	174.04	9.61	286.81	20.87	4.24	24	63.29	10.98	129.78	119.63	7.74	850.98	85.1
Phosphorus (mg)	355.55	35.2	239.31	152.88	58.62	159.47	44.75	11.27	401.46	122.96	12.95	1594.41	199.3
Iron (mg)	3.1	1.58	1.34	2.22	0.27	1.79	2	0.35	6.29	8.83	0.4	28.18	281.8
Zinc (mg)	2.23	0.89	1.39	1.3	0.6	1.14	0.32	0.54	4	1.36	0.31	14.1	94
Magnesium (mg)	48.9	27.85	73.43	94.68	17.26	38.96	28.91	13.38	130.36	56.01	6.47	536.21	141.1
Potassium (mg)	253.97	230.83	369.46	284.52	60.47	353.79	352.8	182.05	741.5	385.23	142.01	3356.63	119.9

Sodium (mg)	536.41	0.41	57.98	8.97	186.31	107.41	253.16	0.4	572.89	548.15	0.41	2272.51	98.8

[a] Carbohydrates, protein, and fat contribute 54.5%, 17.7%, and 28.2% of total energy, separately. Saturated fatty acids were 7.4% of total energy. PMS ratio was 1.6 : 1.1 : 1.

Table S4. Nutrient analysis for the suggested plant-based diet plan B at 2100 Kcal level. [a]

	Breakfast			Lunch					Dinner					
	**Taiwanese spring roll with whole wheat wrap**	**Morning drink made with mung bean & Job's tear**	**Apple**	**Sweet potato and brown rice congee**	**Stewed milkfish**	**Braised mushroom and wheat gluten rolls**	**Stir-fried bell pepper and white water snowflake stems**	**Guava**	**Steamed brown rice**	**Pan-fried oyster and egg with green veggi and minced tomato**	**Braised chicken with chestnut**	**Boiled sweet potato leaves**	**Papaya and Unsweetened yogurt**	**Total**	**DRIs (%)**
Energy (kcal)	479.58	117.51	51.59	246.17	129.67	123.51	49.5	57.86	173.84	197.8	146.95	54.23	233.93	2062.15	98.2
Carbohydrate (g)	60.41	23.88	13.91	51.9	1.83	12.03	3.21	14.86	37.34	13.79	6.6	3.39	40.18	283.33	
Dietary fiber (g)	10.25	2.23	1.35	2.41	0.48	4.78	1.37	5.26	1.42	0.75	1.25	2.54	3.44	37.54	129.4
Protein (g)	25.16	4.36	0.21	4.64	6.68	9.81	0.75	1.03	3.79	13.78	6.85	2.38	8.36	87.79	125.4
Fat (g)	17.41	0.76	0.09	1.7	10.45	4.58	3.97	0.11	0.62	11.04	9.38	3.95	5.41	69.48	
SFA (g)	2.99	0.14	0.02	0.4	3.17	0.77	0.73	0.03	0.12	2.9	1.98	0.5	3.61	17.36	
MUFA (g)	4.78	0.35	0	0.73	4.21	1.02	0.93	0.01	0.28	3.6	3.23	3.03	1.55	23.73	
PUFA (g)	9.57	0.27	0.03	0.57	2.43	2.78	2.31	0.05	0.21	4.46	4.16	0.41	0.2	27.46	
Cholesterol (mg)	0	0	0	0	37.62	0	0	0	0	244.4	30.76	0	23.9	336.68	
Vitamin A (μg RE)	4.95	0.59	1.58	4.03	34.51	0	41.36	10.01	0	165.45	18.81	554.46	128.87	964.62	160.8
Vitamin C (mg)	75.82	1.65	2.91	5.89	1.22	0.99	36.68	195.61	0.08	14.95	4.68	22.87	90.03	453.38	453.4
Vitamin D (μg)	0	0	0	0	3.85	1.52	0	0	0	1.64	0.04	0	0.12	7.17	71.7
Vitamin E (mg α-TE)	2.64	0.54	0.09	0.5	0.58	0.87	1.09	0.31	0.21	2.37	1.01	1.23	0.28	11.71	97.6
Vitamin B1 (mg)	0.33	0.12	0.02	0.28	0.01	0.1	0.01	0.05	0.22	0.11	0.07	0.05	0.14	1.5	125.4
Vitamin B2 (mg)	0.2	0.05	0.01	0.04	0.04	0.28	0.12	0.04	0.04	0.5	0.07	0.1	0.46	1.96	150.6
Niacin (mg)	4.13	0.38	0.05	3.77	1.9	3.1	0.49	1	1.7	1.72	1.94	0.51	0.61	21.29	133
Vitamin B6 (mg)	0.41	0.07	0.04	0.21	0.23	0.13	0.06	0.13	0.07	0.2	0.22	0.14	0.25	2.17	144.9
Vitamin B12 (μg)	0	0	0	0	0.37	0	0	0	0	16.78	0.12	0	0.59	17.87	744.5
Calcium (mg)	464.05	62.97	3.67	16.88	10.78	7.47	14.55	10.5	5.83	112.53	13.8	72.25	257.3	1052.58	105.3
Phosphorus (mg)	433.22	77.19	9.98	195.25	72.89	95.05	15.59	19.71	112.64	206.84	68.5	30.79	204.48	1542.12	192.8
Iron (mg)	8.67	0.94	0.13	1.35	0.69	1.29	1.96	0.2	0.46	5.04	0.66	1.99	0.42	23.8	238
Zinc (mg)	3.66	0.65	0.38	1.36	0.38	1.23	0.36	1.22	1.32	7.83	0.63	0.44	1.7	21.17	141.1
Magnesium (mg)	155.68	45.6	3.36	73.5	14.12	30.09	7.59	8.29	44.91	49.29	14.55	20.15	45.85	512.98	135
Potassium (mg)	766.95	169.9	118.15	243.88	175.43	270.87	162.02	215.28	123.32	392.35	195.64	310.13	640.36	3784.27	135.2
Sodium (mg)	585.72	5.74	1.56	15.25	332	415	253.49	3.04	0.82	286.01	167.96	125.54	93.2	2285.33	99.4

[a] Carbohydrates, protein, and fat contribute 53.7%, 16.6%, and 29.6% of total energy, separately. Saturated fatty acids were 7.4% of total energy. PMS ratio was 1.6 : 1.4 : 1.

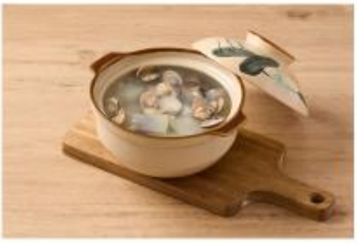

Zinc-rich soup

✓ Clam and winter melon soup, flavored with ginger

Ingredients (4 servings)

Sand spitted clam	400g
Winter melon	600g
Salt	<1g
Sesame oil	10g
Ginger	12g
Water	1000g

Instructions

1. Soak clams in salt water for about 2 hours to remove sand. Rinse and drain.
2. Remove the skin and seeds of the winter melon, and slice the winter melon and shred the ginger.
3. Add sliced winter melon, shredded ginger, and water into a medium sized soup pot and bring to a boil. Cook until winter melon becomes soft and transparent.
4. Add clams and cook until the shells open.
5. Add salt and sesame oil, stir well, turn off heat and serve.

Figure S1. Recipes for "Clam and winter melon soup, flavored with ginger".

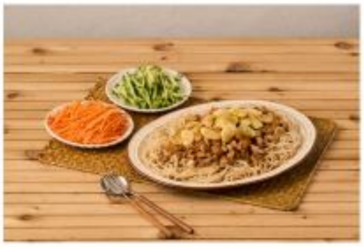

Gluten free dish

✓ Brown rice spaghetti with "stirred fried and fermented soybean paste flavored" hard bean curd cube, chicken cube, and ground pork

Ingredients (4 servings)

Brown rice spaghetti	280g
Lima bean	120g
Chicken	70g
Pork	70g
Hard bean curd	140g
Carrot	100g
Cucumber	100g
Soybean oil	40g
Soy sauce	4g
Fermented bean paste	8g
Sweet bean sauce	16g
White pepper	2g
Garlic	20g
Water	100g

Instructions

1. Dice the hard bean curd, chicken, shred carrot and cucumber, and mince garlic.
2. Prepare a pan, add soybean oil and heat the pan.
3. Stir-fry ground pork, diced chicken, and garlic until the meat change color.
4. Add fermented bean paste, sweet bean sauce, and white pepper, and stir-fry.
5. Add hard bean curd cube and lima bean, and stir-fry.
6. Add water and cook until almost dried-up.
7. Boil a pot of hot water, blanch the carrot and cucumber, and then cook the brown rice spaghetti.
8. Prepare a plate, place brown rice spaghetti in the plate, top with meat sauce, and blanched carrot and cucumber shred, and serve. You may use uncooked carrot and cucumber, if desired.

Figure S2. Recipes for "Brown rice spaghetti with "stirred fried and fermented soybean paste flavored" hard bean curd cube, chicken cube, and ground pork".

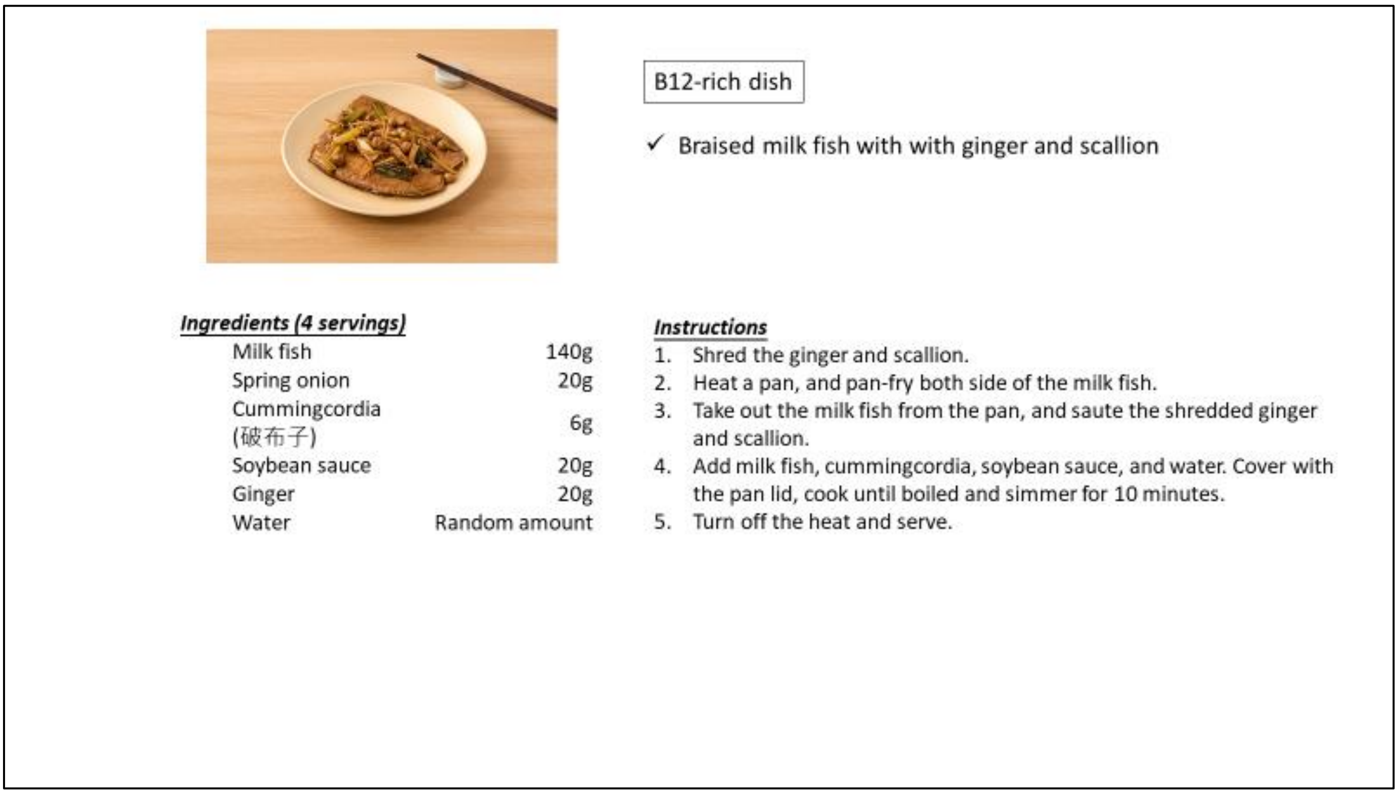

Figure S3. Recipes for "Braised milk fish with ginger and scallion".
